# Diagnostic and prognostic value of lactate metabolism-related genes in Sepsis

**DOI:** 10.1016/j.clinsp.2025.100738

**Published:** 2025-08-06

**Authors:** Guitao Pan, Guiming Huang, Ruiming Deng

**Affiliations:** a Department of Oral and Maxillofacial Surgery, Ganzhou People’s Hospital, Ganzhou City, Jiangxi Province, China; b Department of Anesthesiology, Ganzhou People's Hospital, Ganzhou City, Jiangxi Province, China

**Keywords:** Sepsis, Diagnostic, Prognostic, Lactate metabolism-related

## Abstract

**Objective:**

This study explored the effect of Lactate Metabolism-Related Genes (LMRGs) in the diagnosis and prognosis of Sepsis.

**Methods:**

599 LMRGs were gained via the Molecular Signatures and GeneCards databases. Next, the authorssifted out Differently Expressed LMRGs (DE-S-LMRGs) through differential expression analysis, Protein-Protein Interactions (PPI) networks, and Weighted Gene Co-expression Network Analysis (WGCNA). Then, DE-S-LMRGs were subjected to Least-Absolute Shrinkage and Selection Operator (LASSO), gene expression patterns, Receiver Operating Characteristic (ROC), and Kaplan-Meier (K-M) survival analyses for obtaining diagnostic genes. Subsequently, the authorsyielded independent factors via univariate and multivariate COX analyses. The diagnostic genes expression levels, immune cell infiltration, immune checkpoint, Human Leukocyte Antigen (HLA) molecules, and K-M survival curves between the clusters were compared. Finally, the authorsapplied quantitative Real-Time Polymerase Chain Reaction (qRT-PCR) for verifying the expression of diagnostic genes in the light of normal and Sepsis samples.

**Results:**

Four diagnostic genes (APRT, ARG1, UMPS, and LDHB) were identified, which were mainly concentrated in energy metabolism-related functions and immune-related pathways. In addition, age and ARG1 were selected as independent prognostic factors. The two clusters of GSE65682 datasets show significant differences in the expression, immune cells, immune checkpoints, HLA molecules, and survivability of the four diagnostic genes. qRT-PCR revealed that the expression levels of four diagnostic genes were congruent with the results of bioinformatics analysis.

**Conclusion:**

APRT, ARG1, UMPS, and LDHB might be new ideas for studies related to the diagnosis and treatment of Sepsis.

## Highlights

• Identified 4 diagnostic genes (APRT, ARG1, UMPS and LDHB) for sepsis prognosis.
• ARG1 and age are independent prognostic factors for sepsis outcomes.
• qRT-PCR confirmed diagnostic gene expression patterns in sepsis patients.

## Introduction

Sepsis is a disorder of the immune system resulting in invading pathogens, and can lead to major organ dysfunctions. Sepsis is a common disease in intensive care unit patients, the incidence of which was 437 to 1031 cases per 100,000 people per year.^1^ At present, the diagnosis of Sepsis is usually based on a series of medical tests, including blood, urine, wound secretions, and mucus, but these tests can cause delays in diagnosis and intervention. Currently, clinical treatment of Sepsis includes anti-infection, fluid replacement, and maintenance of organ function.^2^ However, the mortality rate of Sepsis patients with critical illness remains high, and the key factors and mechanisms that determine their prognosis remain unclear.

Lactic acid is the product of anaerobic metabolism during tissue ischemia and hypoxia, and is an important indicator reflecting microcirculation. Lactic acid levels increase significantly when shock occurs in the body.^3^^,^^4^ Further, lactate is a product of anaerobic glycolysis, and elevated levels of lactate are found during stress, hypoxia, and other critical illnesses. Current studies have shown that the accumulation of lactate in Sepsis patients’ bodiesincreases the risk of death, and higher lactate levels are associated with poorer survival outcomes.^4^ Therefore, the lactate level can be an independent prognostic factor in Sepsis patients. Nevertheless, the importance of LMRGs in Sepsis’ diagnosis and prognosis remains unclear.

In current decades, genome-wide analyses, something as high-throughput sequencing technology and gene chips, have been regularly utilized to investigate gene expression profiles.^5^ Consequently, the authorsdesigned to identify the diagnostic value and prognostic significance of LMRG in Sepsis, exploring the immune indexes and survival among Sepsis samples in different clusters grouped by consensus clustering, which would provide new ideas for Sepsis’ diagnosis, treatment and prognosis.

## Material and methods

### Ethical statement

The study protocol was conducted at the Ganzhou People's Hospital from May 2022 to March 2023 in accordance with the Declaration of Helsinki. The study was approved by the ethics committee of The Ganzhou People's Hospital (TY-ZKY2022–032–01) and was registered in the Chinese Clinical Trial Registry (ChiCTR2300067644, Principal investigator: Rui-ming Deng, Date of registration: 2023–1–16). Written informed consent was obtained from all participants in this study.

### Data source

The authorsyielded the GSE65682 and GSE28750 datasets of blood sample transcriptomes data and clinical information for Sepsis samples via the GEO database, with the GSE28750 dataset as an external validation set. GSE65682 dataset contains the Sepsis expression profile data from 760 case samples and 42 control samples, which was generated based on GPL13667[HG-U219] Affymetrix Human Genome U219 Array platform. And there were 10 Sepsis samples and 20 control samples involved in GSE28750. A total of 485 and 284 LMRGs were obtained by searching “Lactate Metabolism-Related Gene” in the GeneCards^6^ (Supplementary Table 1) and Molecular Signatures (Supplementary Table 2) databases,^7^ respectively. Following the merging of the genes obtained from the two databases and deleting the duplicate genes, 599 LMRGs were integrated for further analysis (Supplementary Table 3).

### Screening for DE-S-LMRGs

On the basis of 599 LMRGs, the edgeR (version 3.34.1) was applied to screen the differentially expressed Sepsis LMRGs (DE-S-LMRGs) between the Sepsis and normal samples in the GSE65682 dataset (|log2FC| >1, *p* < 0.05), and the Benjamini-Hochberg (BH) method was applied for correction. The ggplot2 (version 3.3.5)^8^ was applied to draw a volcano plot for displaying the differential expression of genes, and pheatmap (version 1.0.12)^9^ was used to draw a heatmap of differential expression of DE-S-LMRGs in Sepsis samples and controls.

### Expression patterns and biological significance of DE-S-LMRGs

Correlation analysis was performed on DE-S-LMRGs using the Pearson correlation test in the psych package (version 2.1.9). Interactions of DE-S-LMRGs were plotted into a PPI network via the Search Tool for the Retrieval of Interacting Genes (STRING; http://string.embl.de/) database, and the authorssifted out interactive genes (interaction score was greater than 0.4).

The interacting DE-S-LMRGs genes were annotated by KEGG and GO to explore biological significance represented by each gene, and the Benjamini-Hochberg (BH) method was applied for correction. GO and the KEGG pathway enrichment analyses for DE-S-LMRGs (*p* < 0.05) were implemented by the Clusterprofiler package (version 4.0.2).^10^

### Screening for key genes

The WGCNA package (version 1.70–3)^11^ was utilized to construct a network in the GSE65682 dataset to screen for Sepsis-related modules. The authorsconsidered disease state (normal and Sepsis) as the trait data for WGCNA. First, samples were clustered to delete the abnormal samples. According to clinical feature information, sample clusters and clinical feature heatmaps were plotted, and the soft thresholds was determined. Based on the optimal soft threshold, the minimum number of genes in each gene module was set according to the criteria of the hybrid dynamic tree cutting algorithm. Correlation between modules and disease state was computed to sift out key modules. Overlap analysis was implemented on key module genes and DE-S-LMRGs in the PPI network, and the authorsconsidered the intersected genes as key genes.

### Sifting for key genes

The key genes were analyzed using the machine learning LASSO regression algorithm to obtain candidate diagnostic genes. LASSO regression analysis was implemented on the expression of key genes based on sample information (Sepsis and normal) via glmnet (version 4.1–3).^12^

In order to sift out genes with consistent gene expression patterns in the GSE65682 and GSE28750 datasets, box plots of candidate diagnostic genes were drawn. For evaluating the ability of candidate diagnostic genes to assess disease, the pROC (version 1.18.0)^13^ was used to draw ROC curves of candidate diagnostic genes in the GSE65682 training set and GSE28750 Test set, and the candidate diagnostic genes with the AUCs greater than 0.7 were regarded as diagnostic model genes.

Finally, the survminer (version 0.4.9)^14^ was utilized to plot K-M survival curves for high/low-risk groups of diagnostic model genes in the GSE65682 dataset, and genes notably associated with the survival were considered as diagnostic genes.

### Single-gene GSEA analysis of diagnostic DE-S-LMRGs

For further exploring related signaling pathways and underlying biological mechanisms in high/low-risk groups, the authorsutilized clusterProfiler (version 3.18.1)^10^ and org. Hs.eg.db (version 3.12.0) to analyze diagnostic genes. GO and KEGG enrichment analyses of diagnostic genes using the GSEA gene set file (http://www.gsea-msigdb.org/gsea/msigdb) (GO: c5.go.v7.4.entrez.gmt, KEGG:c2.cp.kegg.v7.4.entrez.gmt). The authorsdivided samples into high/low-risk groups based on the median value of individual diagnostic gene expression values, and GSEA was implemented on all genes in high/low-risk groups. Thresholds were |NES| >1, NOM *p* < 0.05 and *p* < 0.25, and the Benjamini-Hochberg (BH) method was applied for correction.

### Single-gene GSEA analysis of diagnostic DE-S-LMRGs

Based on 24 immune cell sets, the ssGSEA algorithm was applied to compute immune cell infiltration of Sepsis and normal samples of the GSE65682 dataset, and the rank sum test was utilized to explore the difference in immune cell infiltration between the Sepsis group and the normal group. Then, ggplot2 (version 3.3.5)^8^ was applied to demonstrate comparison of immune cell scores between controls and Sepsis samples. Additionally, ggplot2 (version 3.3.5)^8^ was used for analyzing difference of immune checkpoints [CD274 (PD-L1), CTLA-4 (CTLA4), LAG-3 (LAG3), LGALS9 (GAL9), HAVCR2 (TIM-3), PDCD1 (PD-1), PDCD1LG2 (PD-1LG2) and TIGHT (TIGIT)] and HLA molecules between the Sepsis and normal samples. Moreover, Pearson correlation analysis for the association of LMRG diagnosis with significantly different immune cells, dramatically different HLA, and significantly different immune checkpoints (*p* < 0.05).

### Immune-related analysis of sepsis

Based on 24 immune cell sets, the ssGSEA algorithm was used to calculate the immune cell infiltration of the Sepsis and normal samples in the GSE65682 dataset, and the Wilcoxon rank-sum test was used to analyze the difference in immune cell infiltration between the Sepsis group and the normal group (*p* < 0.05). The ggplot2 package (version 3.3.5)^13^ was used to show a comparison of immune cell scores between control and Sepsis samples. Additionally, the ggplot2 package (version 3.3.5)^13^ was used to analyse the difference of immune checkpoints [CD274 (PD-L1), CTLA-4 (CTLA4), LAG-3 (LAG3), LGALS9 (GAL9), HAVCR2 (TIM-3), PDCD1 (PD-1), PDCD1LG2 (PD-1LG2) and TIGIT (TIGIT)] and HLA molecules between the Sepsis and normal samples. Moreover, Pearson correlation analysis for the association of LMRG diagnosis with significantly different immune cell subpopulations (*p* < 0.05), significantly different HLA (*p* < 0.05), and significantly different immune checkpoints (*p* < 0.05) was applied.

### Independent prognostic analysis

To investigate the prognosis of clinicopathological characteristics and diagnostic genes, the diagnostic genes, age, gender, and other clinicopathological factors were included in the risk model by univariate COX-independent prognostic analysis in samples containing all clinical information. Factors with *p* < 0.05 in the univariate COX analysis were included in the multivariate COX analysis using the Forestplot (version 2.0.1) plot.^15^ Clinical factors with *p* < 0.05 in a multivariate COX analysis of independent prognosis were used to construct a nomogram using rms (version 6.2–0)^16^ and regplot (version 1.1) packages. To verify the model performance of the nomogram, the ROC curve analysis was performed using timeROC (v 0.4).

### Consistent clustering based on diagnostic DE-S-LMRGs

The Sepsis samples in the dataset GSE65682 were clustered consistently according to the diagnostic genes related to patient survival, and the optimal K value of the number of clusters was determined according to the results of consistent clustering. The Principal Component Analysis (PCA) dimensionality reduction processing on different clustered samples was performed to observe the distribution of different clustered samples. The ggplot2 (version 3.3.5)^8^ was used to draw a boxplot to display the expression levels of diagnosed genes between different clusters.

To explore the activated pathways in different clusters, the Hallmark gene set was downloaded using the msigdbr (version 7.4.1),^17^ and then the ssGSEA analysis was performed based on the Hallmark pathway as the default pathway via the GSVA package (version 1.40.1).^18^

### Difference analysis between different clusters of Sepsis

The ssGSEA algorithm was applied to compute immune cell infiltration of clusters of Sepsis in the GSE65682 dataset, and difference in immune cell infiltration between Sepsis and the normal groups was analyzed via the Wilcoxon rank-sum test (*p* < 0.05), and shown using the ggplot2 package (version 3.3.5).^8^ Likewise, differences in immune checkpoint and HLA molecules were compared in Sepsis clusters, and their expression was plotted using the ggplot2 package (version 3.3.5).^8^ K-M survival curves between Sepsis clusters were drawn via the survminer package (version 0.4.9).^14^

### qRT-PCR

qRT-PCR was applied to verify the expression of diagnostic genes based on the blood samples of 10 normal and 10 Sepsis samples. Samples were approved through the patients and the Ethics Committee of Ganzhou People's Hospital. The authorsextracted total RNA via TRIZol (Thermo Fisher, ShangHai, CN), Following mRNA was reverse transcribed into cDNA, the qPCR reactions was performed using the SureScript-First-strand-cDNA-synthesis-kit (Servicebio, WuHan, CN), and the specific experimental steps were carried out according to the instructions. The primers of the diagnostic markers were displayed in Table 1. The expression of diagnostic genes was calculated using 2^−ΔΔCt^ method, and data were compared by the *t*-test.

**Table 1:** The primer sequences of the diagnostic markers used in qRT-PCR.

Primers		Sequence
APRT	APRT-F	CTTCCCCGACTTCCCCAC
	APRT-R	TCCACGACGACCACCCTC
ARG1	ARG1-F	AACTCGAACAGTGAACACAGCAG
	ARG1-R	GTGGGTTAAGGTAGTCAATAGGC
UMPS	UMPS-F	CTGAGGAGCACTCTGAATTTGTT
	UMPS-R	CTGAGATTATGCCACGACCTACA
LDHB	LDHB-F	CTTGCTCTTGTGGATGTTTTGG
	LDHB-R	CGACTCTCCCCTTCTTGCTGAC
GAPDH	Reference gene-GAPDH F	CGAAGGTGGAGTCAACGGATTT
	Reference gene-GAPDH R	ATGGGTGGAATCATATTGGAAC

## Results

### Screening for DE-S-LMRG

**Fig. 1:**
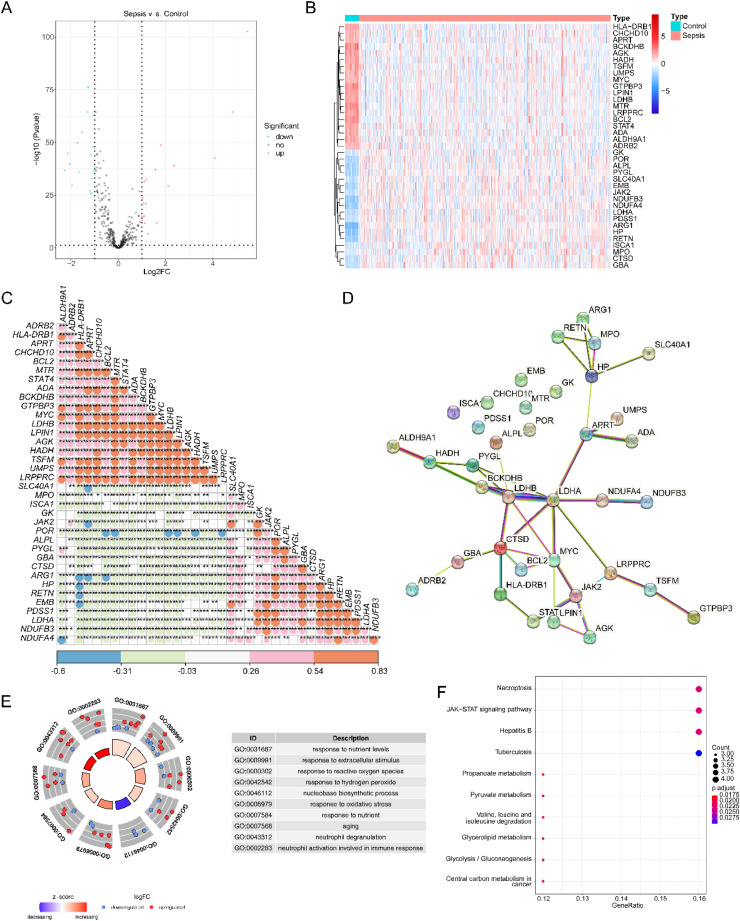
Identification and analysis of DEGs and DE-S-LMRGs in Sepsis and normal samples. (A) Volcano map of DEGs in Sepsis and normal samples. Each dot in the figure represents a gene. The red dots represent up-regulated DEGs, the blue dots represent down-regulated DEGs in Sepsis; (B) Heatmap of DEGs in Sepsis and normal samples. Each row represents the relative expression level of a gene in all samples, each column represents the relative expression level of all genes in a sample, and the color of each square represents the relative expression level of the gene in the sample (blue represents low expression level and red represents high expression level); (C) Correlation diagram of DE-S-LMRGs; (D) PPI network of 30 DE-S-LMRGs; (E) GO enrichment analysis of DE-LMRGs. The outer circle is go term. The dots represent genes. Blue indicates down-regulation genes and red indicates up-regulation genes. The darker the color of the inner circle, the more significant it is. The larger the volume, the more genes are enriched on this pathway; (F) KEGG enrichment analysis of DE-LMRGs. Corresponding pathways of KEGG enrichment in the top 10 of significance were shown. The abscissa is the ratio of genes, the ordinate is the name of the pathway, and the size of the origin represents the number of genes. The color of the pathway is related to significance, and the stronger the significance, the redder the color.

Based on 599 LMRGs, the expression profiles of 385 S-LMRGs were first selected in the GSE65682. Moreover, 37 DE-S-LMRGs were screened using the differential expression analysis, including 18 up-regulated genes and 19 down-regulated genes (Fig. 1A and 1B). Interestingly, the authorsfound high expression of LDHA and low expression of LDHB in patients with Sepsis. Expression patterns and biological significance of DE-S-LMRGs.

With the Pearson correlation analysis among the 37 DE-S-LMRGs, it was shown that there is a significant correlation between most genes. For example, ALDH9A1 (Fig. 1C) was positively correlated with HLA-DRB1, GTPBP3, MYC, etc.; HLA-DRB1 (Fig. 1C) was negatively correlated with ARG1, HP, RETN, etc. Furthermore, a PPI network containing 30 DE-S-LMRGs was established (Fig. 1D). The core genes of PPI were LDHA, MYC, CTSD and LDHB. The LDHA contained 8 edges, including APRT, PYGL, BCKDHB, LDHB, CTSD, MYC, LRPPRC and NDUFA4. MYC contained 6 edges, including LDHA, LDHB, CTSD, BCL2, LPIN1 and JAK2. CTSD contained 6 edges, including LDHB, GBA, HLA-DRB1, BCL2, MYC and LDHA. LDHB contained 6 edges, including ALPL, PYGL, BCKDHB, CTSD, MYC and LDHA.

Function enrichment analysis revealed that the 30 DE-S-LMRGs of the PPI network enriched 191 GO-Biological Process (GO-BP), 27 GO—Cellular Component (GO—CC), 11 GO-Molecular Function (GO-MF), and 146 KEGG pathways. These GO terms were major included lumen and membrane, biological response, energy metabolism (Fig. 1E). Additionally, these KEGG pathways were related to energy metabolism, such as propanoate metabolism, pyruvate metabolism, and glycerolipid metabolism (Fig. 1F).

### Screening for DE-S-LMRGs by WGCNA

**Fig. 2:**
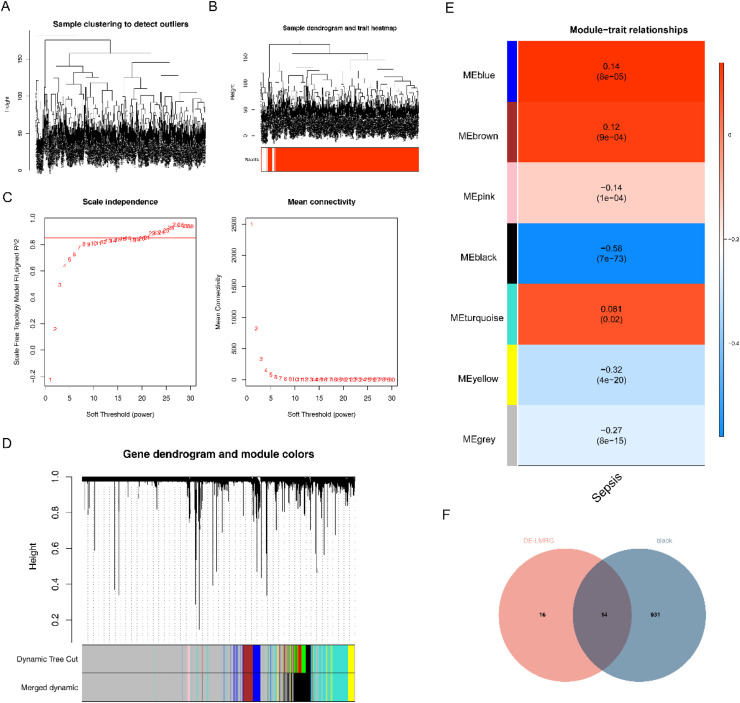
Screening for DE-S-LMRGs by WGCNA. (A) Sample clustering diagram. The branch represents the sample, and the ordinate represents the height of the hierarchical clustering. There is no obvious outlier sample; (B) Sample and trait clustering plots; (C) Analysis of network topology for various soft-thresholding powers, including the scale-free fit index (y-axis of left panel) and the mean connectivity (degree, y-axis of right panel). The higher the square of the correlation coefficient is R^2, the closer the network is to a scale-free distribution; (D) Module clustering dendrogram. Above is the hierarchical cluster tree of genes; below is the gene module. Corresponding genes clustered in the same branch are divided into the same module, with different colors representing different modules; (E) Heatmap of the relationship between gene modules and traits in Sepsis. Red is positive correlation and blue is negative correlation. The number in the cell represents correlation and significance; (F) Venn diagrams of key genes.

To obtain Sepsis-related genes, the WGCNA was performed on the GSE65682 dataset. The clustering of GSE65682 dataset was no filter samples (Fig. 2A), and the clustering effect of patient samples and normal samples was better (Fig. 2B). When the R^2 equal to 0.85, the optimal soft threshold equal to 21 were selected (Fig. 2C). Furthermore, minimum number of genes per gene module was set to 100, then 9 modules were acquired. In addition, the authorsyielded 7 modules after merging (MEDissThres = 0.1) (Fig. 2D). The Black module (R^2 = −0.58, *p* = 7e-73) was significantly negatively correlated with Sepsis, and the black module was identified as a key gene module, including 945 module genes (Sepsis-related genes) (Fig. 2E). Finally, 14 key genes were obtained from the overlap analysis between 945 Sepsis-related genes and 30 DE-S-LMRGs (Fig. 2F).

### Four diagnostic DE-S-LMRGs were selected by analysis

**Fig. 3:**
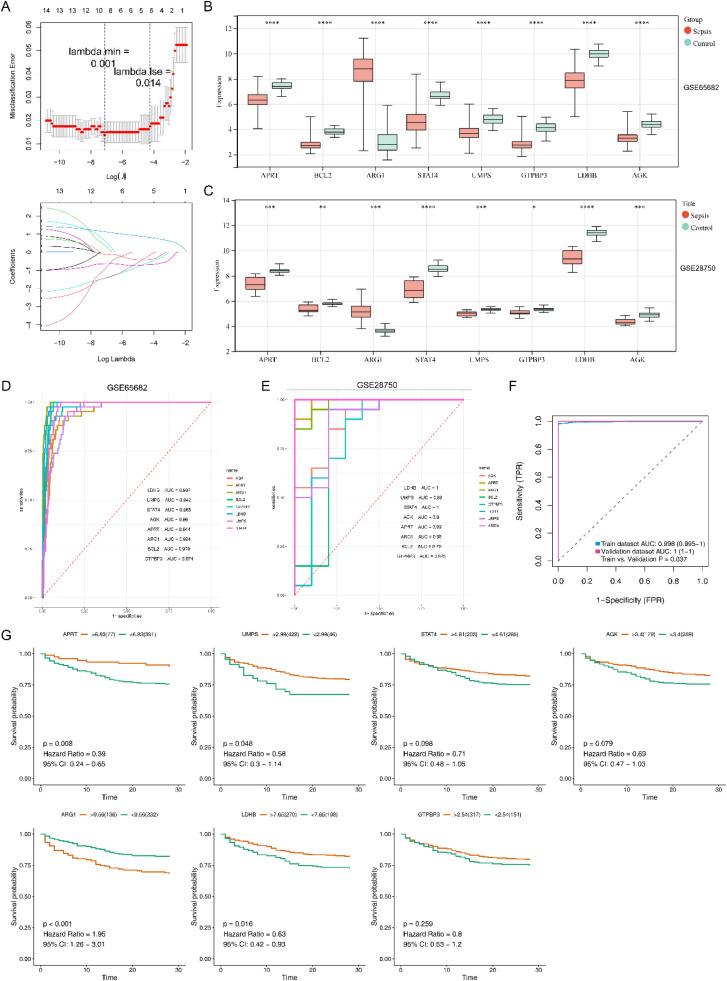
Four diagnostic DE-S-LMRGs were selected. (A) Four diagnostic DE-S-LMRGs were selected by LASSO regression analysis. Up (Error diagram for cross validation): The abscissa is log (Lambda), and the ordinate represents the error of cross validation; down (Graph of gene coefficients): the abscissa is log (Lambda) and the ordinate is the coefficient of the gene (left); (B) Boxplot for the expressions of hub genes in GSE65682; (C) Boxplot for the expressions of hub genes in GSE28750; (D) ROC curve for eight diagnostic model genes in GSE65682 (test set); (E) ROC curve for eight diagnostic model genes in GSE28750 (validation set); F, ROC curve of the diagnostic model in GSE65682 and GSE28750; (G) KM survival curves of diagnostic genes in patients with Sepsis in GSE65682.

A total of 8 candidate diagnostic genes were selected by LASSO (AGK, APRT, ARG1, BCL2, GTPBP3, LDHB, UMPS, and STAT4) (Fig 3A). Fig. 3B and Fig. 3C displayed that ARG1 expression was upregulated in the Sepsis samples from GSE65682 and GSE28750 datasets, and the expression of the other seven candidate diagnostic genes was significantly lower in the Sepsis samples than in the normal samples. Furthermore, 7 diagnostic model genes (AGK, APRT, ARG1, GTPBP3, LDHB, UMPS, and STAT4) with AUC greater than 0.8 in both datasets (GSE65682 dataset and GSE28750 dataset) were regarded as a diagnostic model (Fig. 3D and 3E). And meanwhile, the AUCs of this diagnostic model were greater than 0.8 in both datasets (GSE65682 dataset and GSE28750 dataset) (Fig. 3F). Among 7 diagnostic model genes, 4 genes (APRT, ARG1, UMPS and LDHB) were sensibly related to survival of patients, and survival of patients in high/low expression groups was also significantly different, which were further considered final diagnostic genes (Fig. 3G).

### Single-gene GSEA enrichment analysis of diagnostic DE-S-LMRGs

**Fig. 4:**
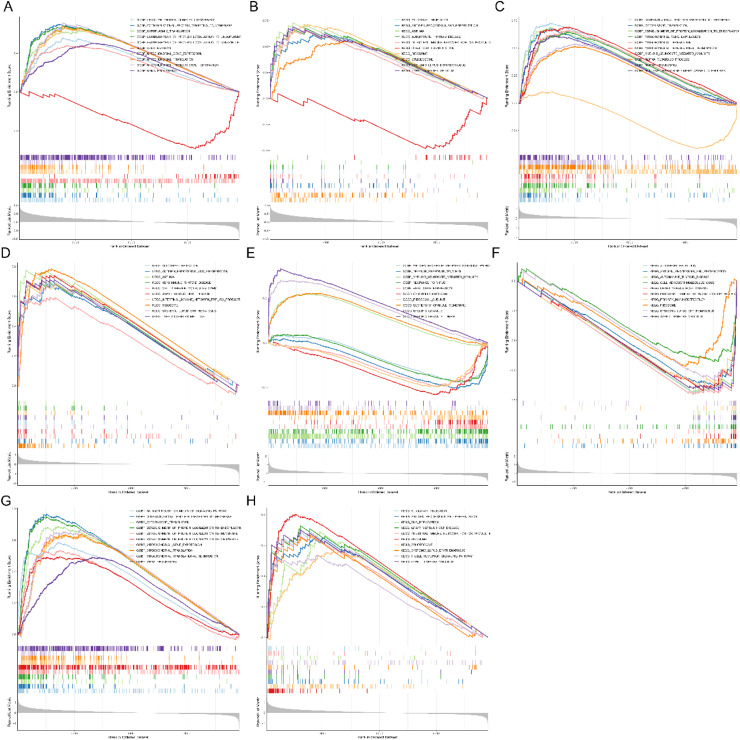
Single-gene GSEA enrichment analysis of diagnostic DE-S-LMRGs. (A) UMPS GO enrichment analysis; (B) UMPS KEGG enrichment analysis; (C) APRT GO enrichment analysis; (D) APRT KEGG enrichment analysis; (E) ARG1 GO enrichment analysis; (F) ARG1 KEGG enrichment analysis; (G) LDHB GO enrichment analysis; (H) LDHB KEGG enrichment analysis.

In order to provide greater insight targeting the biological significance of four diagnostic genes, single-gene GSEA analysis was conducted. It could be seen that UMPS was mainly enriched in the GO terms of COTRANSLATIONAL PROTEIN TARGETING TO MEMBRANE and the KEGG pathways of ASTHMA (Fig. 4A and 4B); APRT was enriched in 313 GO terms and 27 KEGG pathways, such as COTRANSLATIONAL PROTEINTARGETINGTO MEMBRANE and RIBOSOME (Fig. 4C and 4D); ARG1 was related to the terms of SRECIFIC GRANULE LUMEN and the signal pathway of GRAFT VERSUSHOST DISEASE (Fig. 4E and 4F); LDHB was enriched in 437 GO terms and 23 KEGG pathways (Fig. 4G and 4H).In general, the GO terms were mainly related to energy metabolism, such as mitochondrial gene expression, mitochondrial translation, and mitochondrial translational termination. The KEGG pathways were mainly related to immune, containing allograft rejection, graft versus host disease, and primary immunodeficiency, etc.

### Immune-related analysis of Sepsis

**Fig. 5:**
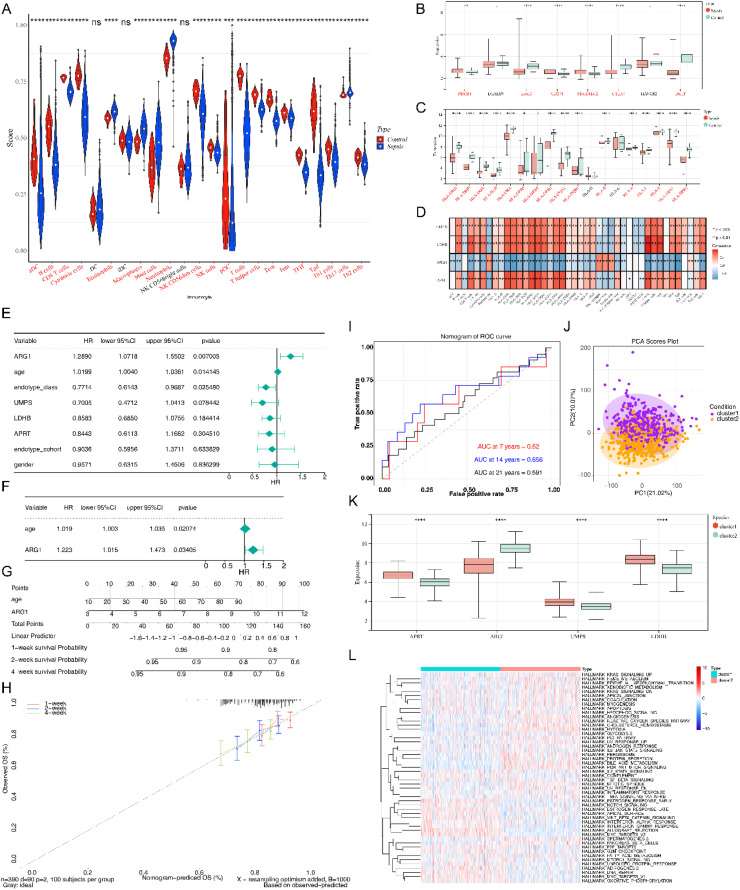
Comprehensive analysis of diagnostic DE-S-LMRGs and the two diagnostic DE-S-LMRGs-based clusters were generated. (A) Differences in immune cell infiltration between Sepsis and normal samples in the GSE65682 dataset; (B) Differences in immune checkpoints between Sepsis and normal samples in the GSE65682 dataset; (C) Differences in leukocyte antigen molecules between Sepsis and normal samples in the GSE65682 dataset; (D) Diagram of correlation between diagnostic genes and immune index; (E) Forest plot for independent prognostic - univariate COX analysis; (F) Forest plot for independent prognostic ‒ multivariate COX analysis. Hazard Ratio (HR) is the risk ratio, and the lower 95 % CI and the upper 95 % CI are the 95 % Confidence Intervals of risk values; (G) Nomogram was constructed based on age and ARG1 (C-index = 0.593). Different risk scores in the nomogram correspond to a survival score, which predicts the 1-, 2-, 4-week survival rate based on the total score; (H) Calibration curve of the nomogram; (I) The ROC curve of nomogram; (J) Consistent clustering results to identify the optimal clusters based on diagnostic DE-S-LMRGs; (K) PCA plots of two clusters of samples; (L) Differences in diagnostic genes expressions between the two clusters of samples; (M) Clustering heatmap between ssGSEA score and enrichment pathway.

From the perspective of the immune microenvironment in Sepsis, except DC, iDC and NK CD56bright cells, the contents of other 20 immune cell subpopulations were markedly different between Sepsis samples and control samples (Fig. 5A). The expression of six immune checkpoints (Fig. 5B) and 16 HLA molecules (Fig. 5C) were notably different between Sepsis and normal samples as well. Additionally, as can be seen in Fig. 5D, four diagnostic genes (APRT, ARG1, UMPS and LDHB) were significantly related to these differentially expressed immune cell subpopulations, differentially HLA, and differential expressed immune checkpoints.

### Age and ARG1 were selected as independent prognostic factors for Sepsis

To investigate the prognosis of clinicopathological characteristics and diagnostic genes, an independent prognostic analysis was performed. Univariate COX independent prognostic analysis indicated that the p-value of ARG1, age and endotype class were screened (Fig. 5E). Furthermore, age and ARG1 were selected as independent prognostic factors by multivariate COX analysis (Fig. 5F). Finally, nomogram with a C-index equal to 0.593 was constructed with age and ARG1, indicating that this nomogram has better predictive ability (Fig. 5G and 5H). The AUC values of the ROC curves of the nomogram at 7, 14, and 21 days all reached above 0.6 (Fig. 5I). This indicates that the nomogram model has a certain predictive ability.

### The samples of Sepsis were divided into 2 clusters based on diagnostic DE-S-LMRGs

Consistent clustering of Sepsis samples in GSE65682 dataset was conducted based on four diagnostic genes (APRT, ARG1, UMPS, LDHB) associated with patient survival. According to the results in Fig. 5J, the optimal number of clusters K was determined to be 2, hence, two clusters of Sepsis were generated. Following the PCA dimension reduction process was performed on the two types of samples, the distribution of the two types of samples was more obvious (Fig. 5K). In addition, the expression of four diagnostic genes (APRT, ARG1, UMPS, LDHB) was significantly different between cluster1 and cluster2 (Fig. 5L). APRT, UMPS and LDHB were significantly highly expressed in the cluster1. ARG1 was significantly highly expressed in cluster2. Moreover, the ssGSEA enrichment scores with the help of the Hallmark gene set were different between the cluster1 and cluster2 (Fig. 5M).

### Immune-related analysis between 2 different clusters of Sepsis

**Fig. 6:**
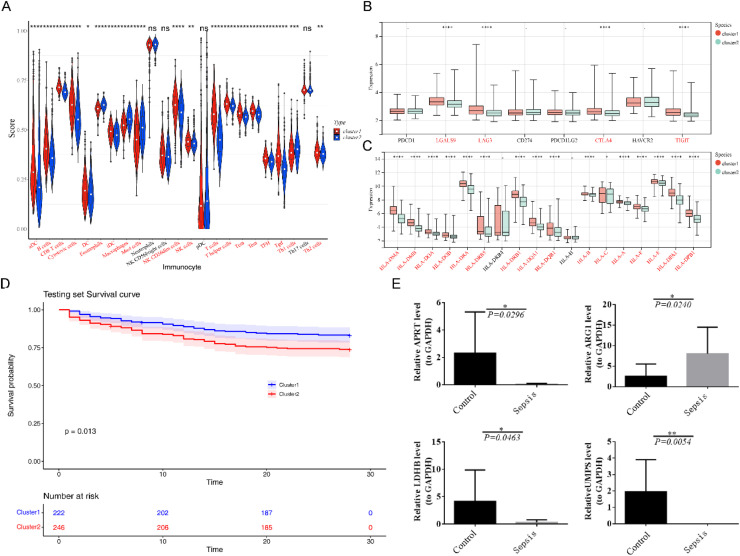
Differential immune cell subpopulations, differential immune checkpoints and differential HLA molecules were investigated between the two clusters of Sepsis. (A) Differences in immune cell infiltration between two clusters of Sepsis; (B) Differences in immune checkpoints between two clusters of Sepsis; (C) Differences in leukocyte antigen molecules between two clusters of Sepsis. For A‒C, the statistical test was conducted using the Wilcoxon rank-sum test; (D) KM survival curves between two clusters of Sepsis (*p* = 0.013); (E) The qRT-PCR was performed to explore the expression differences of diagnostic genes between Sepsis and healthy samples, and the statistical test was conducted using *t*-test.

To understand differences in immune infiltration between clusters, differential immune cell subpopulations, differential immune checkpoints and differential HLA molecules were investigated. Except neutrophils, NK CD56bright cells, pDC and Th17 cells, 20 immune cell subpopulations were notably different between cluster1 and cluster2 (Fig. 6A). Four immune checkpoints (Fig. 6B) and 16 HLA molecules (Fig. 6C) were significantly different between the cluster1 and cluster2. The viability of cluster2 is significantly lower than that of cluster1 (Fig. 6D).

### qRT-PCR

Expression levels of the four diagnostic genes shown in qRT-PCR results were concordant with the results of bioinformatics analysis. These four diagnostic genes were significantly different between the normal and Sepsis samples. Of the four diagnostic genes, except ARG1, all other genes were notably higher in normal samples than in the other one (Fig. 6E).

## Discussion

Sepsis can lead to multiple organ dysfunction and produce chronic and critical disease states marked by severe immune dysfunction and catabolism.^19^ In clinical practice, the Sequential Organ Failure Assessment (SOFA) score is now considered the gold standard for identifying organ dysfunction in Sepsis patients.^20^ However, the early diagnosis and prognosis of Sepsis patients are unclear. Biomarkers of the diagnosis of Sepsis could enable early intervention possible, which can reduce the risk of death. Biomarkers such as CRP, PCT, IL-6, IL-8, CD64, and CD11b, are of great significance for early (24‒48 h) diagnosis of neonatal Sepsis and monitoring the effect of antibiotic treatment before bacterial culture detection results.^21^ Lactate is the most commonly used biomarker to identify Sepsis, which has great importance in improving early diagnosis and prognosis of Sepsis.^22^ Nevertheless, the research on the early diagnosis and prognosis of Sepsis associated with the lactate metabolism gene is still unclear, and expression of lactate metabolism-related genes is earlier than lactate production, so it may have more clinical value in early diagnosis and prognosis than blood lactate.

The opening of the GEO database has provided the convenience for the research progress of the diagnosis and treatment of Sepsis. In the study, inspired by previous research,^23^ the GSE65682 and GSE28750 datasets were selected to screen the potential diagnostic LMRGs in Sepsis, in which a large number of samples in the GSE65682 cohorts served as the training set ensuring the reliability of the results. Hence, four diagnostic genes (APRT, ARG1, UMPS, and LDHB) were further obtained through LASSO, expression analysis, and other data analysis. Among them, APRT deficiency can lead to serious kidney diseases, such as kidney stones, interstitial nephritis, and 2,8-Dihydroxyadenine (DHA) precipitation in renal interstitial failure.^28^ Nucleotide synthesis in red blood cells can only be achieved through the rescue pathway, while APRT enables the repeated utilization of purine bases (hypoxanthine et al.) to convert them into single nucleotides (AMP et al.).^24^ Furthermore, Uridine Synthase (UMPS) not only has a significant role in cell growth and metabolism, but also is a key enzyme involved in uridine biosynthesis, which catalyzes the conversion of orotate to uridylate. As the main pyrimidine nucleotide, uridylic acid is the precursor for the synthesis of other pyrimidine nucleotides. Pyrimidine nucleotides are involved in the maintenance of cell growth and metabolism, and are the structural components of Nucleic Acids (DNA, RNA) and several metabolic factors, which are necessary for glycogen and cell membrane precursor synthesis, glycosylation of proteins and lipids, and glucoaldehyde acidification.^25^^,^^26^

Lactate Dehydrogenase (LDH) consists of two major subunits, LDHA and LDHB, and reversibly catalyzes the conversion of pyruvate to lactate or lactate to pyruvate. LDHA has a high affinity for pyruvate and is a key enzyme in glycolysis, which can promote the conversion of pyruvate to lactate. LDHB has a high affinity for lactic acid and is a key enzyme in the glycolysis pathway, which can promote the conversion of lactic acid into pyruvate.^27^ The authorsfound high expression of LDHA and low expression of LDHB in Sepsis patients. LDHA is responsible for the conversion of pyruvate to lactate and NAD+, while LDHB is responsible for the conversion of lactate to pyruvate and promotes oxidative metabolism,^28^ which suggests that hyperlactemia is an independent predictor of Sepsis death. Promoting LDHB expression and activity may be a potential therapeutic method for reducing hyperlactemia and treating Sepsis.

Sepsis is a serious complication of infection, and immune dysfunction is considered to be an important factor in the occurrence and development of Sepsis. ARG1 protein is released from the body and maintains very high activity in granulocytes. The intercellular space is relevant to inflammation and has a strong inhibitory effect on immunity. Arginase has been proven to damage the functional chain of T-cells by down-regulating the expression of CD3ζ and ε (which are the key components of the T-Cell Receptor (TCR) signal transduction), leading to immunosuppression and poor prognosis and death in Sepsis patients.^29^ Arginine or arginase 1 inhibitors (such as CB-1158) restore T-cell proliferation, and the high arginase activity in dendritic cells downregulates the MHC Class II influence antigen presentation process.^30^

Meanwhile, an increasing number of evidence has shown that macrophages can engulf foreign microorganisms and present antigens, play an important role in physiological processes such as innate immunity, adaptive immune response, regulation of host inflammation, and maintenance of homeostasis through the synthesis and secretion of various cytokines in Sepsis. Impaired or abnormal immune function caused by impaired energy metabolism of macrophages is closely related to the occurrence and development of Sepsis.^19^ Mitochondria are the main organelles of energy metabolism, and their dysfunction is directly involved in the abnormal response of immune cells.^31^ These results suggest that ARG1 is negatively correlated with immune function. Additionally, ARG1 is closely associated with vascular dysfunction. The high expression of ARG1 can consume a large amount of L-arginine and interfere with eNOS activity, which will affect NO synthesis in the eNOS pathway of arginine and lead to vasodilatory dysfunction in different stages of Sepsis.

In the late stage of Sepsis, macrophages switch from M1 type to M2 type and exhibit anti-inflammatory effects. ARG1 is one of the main markers of the macrophage M2 type. It was found that patients with high expression of ARG1 in Sepsis patients had a poor prognosis, which is coincides with this. The authorsfurther verified high expression of ARG1 in the peripheral blood of Sepsis patients by qRT-PCR. All of this information indicates that ARG1 has potential as a biomarker for the accurate diagnosis and prediction of Sepsis.

In the process of GSEA results of the four diagnostic genes (APRT, ARG1, UMPS, and LDHB) suggested that these four diagnostic genes were mainly enriched in mitochondrial gene expression, primary immune deficiency, other energy metabolism-related functions, and immune-related pathways. Sepsis is a serious complication of infection, microcirculation disorders, energy metabolism changes, and immune dysfunction are important factors in the progression of Sepsis. The current study shows that changes in immune function are significantly related to cell metabolism and microenvironment. When mitochondrial function is impaired and hyperlactemia occurs, immune cell function can be inhibited, further aggravating the condition. The metabolic regulatory role of lactate, as the end product of glycolysis, has garnered increasing attention, particularly in the context of sepsis. In this condition, hyperlactatemia serves not only as a marker of tissue hypoxia but also as a significant driver of immune cell dysfunction. Research indicates that lactate exacerbates the immunosuppressive state in sepsis by directly modulating the metabolic reprogramming and epigenetic modifications of immune cells.^23^ Specifically, the accumulation of lactate promotes the polarization of macrophages towards the M2 anti-inflammatory phenotype by stabilizing Hypoxia-Inducible Factor-1α (HIF-1α).^32^ Furthermore, lactate-induced histone lactylation modifications, such as H3K18la, can further activate the transcription of anti-inflammatory genes, thereby impairing the pathogen-clearing capacity of macrophages.^33^ The high lactate environment inhibits the T-Cell Receptor (TCR) signaling pathway by activating the G Protein-coupled Receptor (GPR81), which leads to a reduction in IFN-γ secretion.^34^ Additionally, Lactate accelerates neutrophil apoptosis and reduces Reactive Oxygen Species (ROS) generation by acidifying the intracellular environment, consequently resulting in a decreased capacity for pathogen clearance.^35^ The role of lactate is particularly pronounced in the tumor microenvironment and chronic inflammation, where it significantly affects immune cell function and promotes disease progression. The lactate metabolism-related genes identified in this study serve as novel biomarkers for sepsis, demonstrating distinct advantages over existing clinical standards. Unlike the SOFA score, these gene biomarkers are not influenced by subjective clinical parameters, such as blood pressure and consciousness level, and they can reflect the underlying mechanisms of immune metabolic dysregulation.^36^ For instance, patients exhibiting high ARG1 expression are more susceptible to T-cell exhaustion^37^ a characteristic that the SOFA score fails to identify.

The four diagnostic genes significantly correlated with these corresponding immune indicators. These findings can more fully indicate that the immune microenvironment is critical in Sepsis. Especially with the deepening of the understanding of immune paralysis in the late stage of Sepsis, the treatment of immune regulation of Sepsis has changed in recent years.^38^ Animal experiments and clinical studies have shown that immune-enhancing therapy can improve the immune status of patients and eliminate pathogenic microorganisms, thereby reducing the incidence of acquired infections and reducing mortality. Univariate and multivariate COX analysis based on four diagnostic genes and clinicopathological factors showed that the ARG1 gene and age were independent prognostic factors of Sepsis. In this study, age was used as an independent prognostic factor; it can fully consider the compensatory capacity of the body and provide more accurate prognostic analysis. Studies have shown that ARG1 is a kind of promising biological marker in the diagnosis of Sepsis and prognosis.^39^ The diagnosis and death prognosis of Sepsis in the first week after burn are negatively correlated with age.^40^ These studies also indicate that ARG1 and age can affect the prognosis of Sepsis. However, specific quantification was not carried out. In this study, quantification was accomplished by constructing a nomogram from 1 week to 4 weeks, and the 1‒4-week survival rate of patients with Sepsis could be predicted in light of the total score. Moreover, We recognize that the current C-index of the model is 0.593, which falls into the moderate range. This may be attributed to several factors. Firstly, although the included predictive factors (ARG1, age) are statistically significant, their overall predictive capability still has room for improvement. Secondly, the linear assumptions of the Nomogram limit its ability to capture nonlinear relationships, which may adversely affect prediction accuracy. Despite the need for improvement in the model's predictive performance, the visual scoring of the Nomogram provides a rapid bedside assessment tool. In future research, integrating additional dimensions of biomarkers, such as inflammatory factors and metabolomics indicators, will help enhance the model's discriminative ability. Furthermore, we will introduce machine learning algorithms, such as random forests and neural networks, to address the issue of nonlinear interactions among variables. Finally, we plan to conduct multicenter prospective studies to validate the clinical applicability of this model.

Finally, four diagnostic genes were used for consistent clustering of Sepsis samples, and the optimal number of clusters was 2. PCA results of the two cluster samples were good. There were significant differences in diagnostic genes between the two cluster samples, and the immune index of Cluster1 was notably higher than that of Cluster2. The viability of Cluster2 is significantly lower than that of cluster1. Nevertheless, we also have certain limitations. In this study, firstly, the association analysis faces challenges in establishing a causal relationship between genes and sepsis, as it is susceptible to confounding factors, including environmental influences. Notably, the prognostic predictive function of the ARG1 gene for sepsis lacks adequate in vitro and in vivo experimental validation, which limits the clinical applicability of the related research findings. Secondly, the small sample size and insufficient validation experiments fail to comprehensively represent the variations in patient characteristics, thereby affecting the generalizability and reliability of the results. Additionally, this study did not integrate multi-omics data, such as proteomics and metabolomics, which restricts a holistic understanding of the molecular mechanisms underlying the disease. It is particularly important to highlight that batch effects may exist in the study, especially when utilizing multi-batch data or when experimental conditions are not entirely consistent, potentially impacting the reliability of the results. Future research should elucidate the gene action pathways through functional validation experiments, such as gene knockout and overexpression, conduct multi-center, large-sample studies to enhance the validation process, and adopt multi-omics joint analysis. Simultaneously, it is crucial to standardize data collection criteria across multi-center cohorts, establish a complementary system to compare existing diagnostic standards, and leverage big data and machine learning technologies to optimize models, thereby addressing the challenges of multi-omics data integration and facilitating the efficient clinical translation of sepsis research.

## Conclusion

Four diagnostic genes (APRT, ARG1, UMPS and LDHB) with excellent diagnostic value were identified, which might be mainly concentrated in energy metabolism-related functions and the immune-related pathway in the Sepsis process. For the potential diagnostic performance in clinical utilization, ARG1 and age were selected as independent prognostic factors, and a corresponding nomogram was constructed. Furthermore, two clusters of Sepsis with significant survival differences were generated based on the key diagnostic genes, and immune-related analysis in different clusters was explored.

## Ethics approval and consent to participate

Study protocol was conducted at the Ganzhou People's Hospital from May 2022 to March 2023 in accordance with the Declaration of Helsinki. The study was approved by the ethics committee of The Ganzhou People's Hospital (TY-ZKY2022–032–01) and was registered in the Chinese Clinical Trial Registry (ChiCTR2300067644, Principal investigator: Rui-ming Deng, Date of registration: 2023–1–16). Written informed consent was obtained from all participants in this study. This study follows the STROBE Statement.

## Consent for publication

Written informed consent was obtained from all participants in this study.

## Conflicts of interest

The authors declare no conflicts of interest.

## Appendix Supplementary materials

Image, application 1

Image, application 2

Image, application 3

Image, application 4
