# Supplementary material for: Diagnostic and prognostic value of lactate metabolism-related genes in Sepsis
Source: Clinics (Sao Paulo). 2025 Aug 6;80:100738. doi: 10.1016/j.clinsp.2025.100738 (PMC12351345; doi:10.1016/j.clinsp.2025.100738)
Supplement: Supplementary file 1 [file mmc1.docx]

**CLINICS-D-25-00087**

**Supplementary Table 1‒3**

**Supplementary Table 1** Lactate metabolism-related genes in the Genecards.

**Supplementary Table 2** Lactate metabolism-related genes in the molecular signatures databases.

**Supplementary Table 3** Overlapping genes related to “Lactate Metabolism” identified in the GeneCards and molecular signatures databases.
